# Evaluating Reproducibility and Best Practices for Replicate Design in G-Quadruplex ChIP-Seq Studies

**DOI:** 10.3390/ijms26199769

**Published:** 2025-10-07

**Authors:** Ke Xiao, Rongxin Zhang, Jing Tu

**Affiliations:** 1 State Key Laboratory of Digital Medical Engineering, School of Biological Science and Medical Engineering, Southeast University, Nanjing 211189, China; kexiao@seu.edu.cn; 2 Robert Lurie Comprehensive Cancer Center, Department of Obstetrics and Gynecology, Feinberg School of Medicine, Northwestern University, Chicago, IL 60611, USA

**Keywords:** G-quadruplex (G4), reproducibility estimation, performance comparison, replicate design

## Abstract

G-quadruplex (G4) ChIP-Seq data are critical for studying the roles of G4 structures in various biological processes, yet their reproducibility remains systematically uncharacterized. In this study, we evaluated the consistency of in vivo G4 peaks across multiple replicates in three publicly available datasets. We observed considerable heterogeneity in peak calls, with only a minority of peaks shared across all replicates. To address this challenge, we compared three computational methods—IDR, MSPC, and ChIP-R—for assessing reproducibility and found that MSPC is the optimal solution in reconciling inconsistent signals in G4 ChIP-Seq data. We further demonstrated that employing at least three replicates significantly improved detection accuracy compared to conventional two-replicate designs, while four replicates proved sufficient to achieve reproducible outcomes, with diminishing returns beyond this number. Moreover, we showed that the reproducibility-aware analytical strategies can partially mitigate the adverse effects of low sequencing depth, though they do not fully substitute for high-quality data. Based on our findings, we recommend 10 million mapped reads as a minimum standard for G4 ChIP-Seq experiments, with 15 million or more reads being preferable for optimal results. Our study provides practical guidelines for experimental design and data analysis in G4 studies, emphasizing the importance of replication and robust bioinformatic strategies to enhance the reliability of genome-wide G4 mapping.

## 1. Introduction

G-quadruplexes (G4s) are non-canonical secondary structures formed by guanine-rich (G-rich) nucleic acid sequences [1]. Their fundamental structural unit is the G-tetrad, a planar arrangement of four guanines held together by Hoogsteen hydrogen bonding. Stacking of multiple G-tetrads, stabilized by central monovalent cations, gives rise to the mature G4 structures.

The distribution of DNA G4s in the genome is not random; they are preferentially enriched in functionally significant regions such as telomeres, gene promoters, and 5′ untranslated regions (5′ UTRs) [1]. This distinctive localization pattern suggests that G4s play crucial roles in fundamental cellular processes, including the regulation of gene expression and the maintenance of genomic stability. Consequently, G4s have emerged as highly promising therapeutic targets, particularly in oncology. Precise genome-wide mapping of in vivo G4 structures is therefore essential for elucidating their biological functions and facilitating the development of novel therapeutics.

While motif-based algorithms [2,3] can predict putative G4s (pG4s) and in vitro sequencing methods [4,5] can detect experimentally observed G4s (oG4s) formed in extracellular environment, the dynamic and polymorphic nature of these structures presents unique challenges for their accurate localization in living cells [6]. The formation and resolution of G4s are dynamic processes influenced by cellular activities such as transcription and replication, leading to substantial heterogeneity in G4 landscapes across individual cells within a population. This biological variability introduces inherent noise between replicate experiments, posing a significant challenge for the reliable profiling of in vivo formed G4 structures.

Endogenously formed G4s (eG4s) can be captured in vivo using antibody-based techniques with high-affinity probes antibodies as BG4 or G4P [7,8,9,10]. The majority of eG4 maps have been generated through ChIP-Seq or CUT&Tag experiments in various cell lines. However, since recent concerns regarding the biased distribution of sequencing reads in CUT&Tag data have been raised [11], this study focuses exclusively on G4 ChIP-Seq datasets.

Given the inherent noise described above, a critical yet unaddressed question for these intracellular G4 data is the consistency of peak calls—genomic intervals identified based on sequencing read enrichment—across biological replicates. To address this gap, robust methods for assessing reproducibility are required. Several computational approaches exist for this purpose: The Irreproducible Discovery Rate (IDR) evaluates reproducibility by measuring the consistency of peak rankings between replicate pairs [12]; Multiple Sample Peak Calling (MSPC) integrates evidence from multiple replicates to rescue weak but consistent peaks by combining *p*-values [13]; and ChIP-R employs a rank-product test to statistically evaluate the reproducibility of peak intervals across numerous replicates [14]. Nevertheless, the suitability of these methods for G4 ChIP-Seq data remains to be validated.

In this study, we utilize three G4 ChIP-Seq datasets, each comprising more than two replicates, to evaluate data consistency and test the performance of different reproducibility estimation methods. We first assess inter-replicate concordance by examining the size of peak sets that are supported by different numbers of replicates, the genomic distribution of peaks, and their overlap with pG4s and oG4s. Based on this analysis, we establish a pseudo-gold standard benchmark to evaluate the applicability of the three reproducibility algorithms. Our findings indicate that a strategy based on MSPC is the optimal solution in reconciling inconsistent signals in G4 ChIP-Seq data. We subsequently employ this method to determine the optimal number of replicates and necessary sequencing depth required for robust detection.

## 2. Results

### 2.1. Inconsistency of Peaks Across Replicate Samples

**Figure 1:**
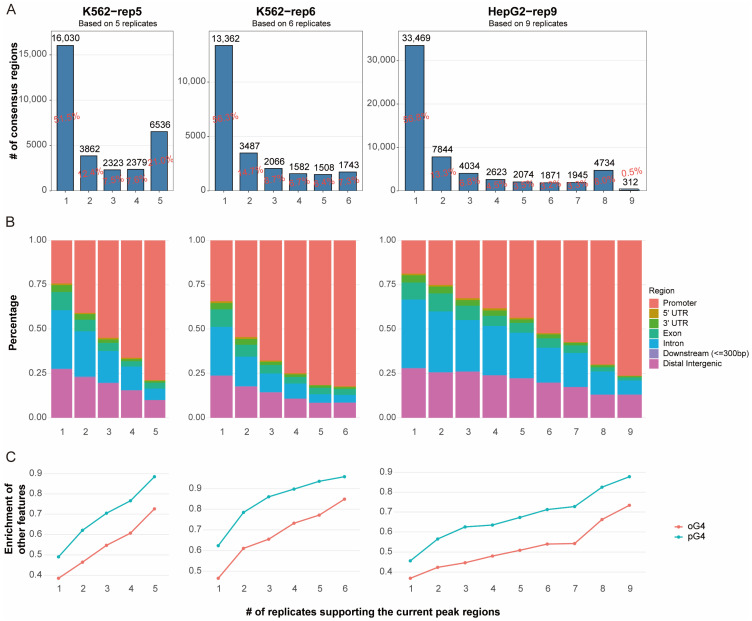
Summary of the G4 peaks from the three datasets. (**A**) Proportion of consensus regions (peaks) supported by k out of N replicates, where k=1,2,…,N, and N is the amount of replicate samples. (**B**) Genomic distribution of peaks identified in k replicate(s). (**C**) Overlap with external G4 annotations (pG4 and oG4 sites) for peaks detected in k replicate(s). The left, middle and right panels correspond to K562-rep5, K562-rep6, and HepG2-rep9, respectively. All available biological replicates within each cohort were included in the analysis.

We assessed the reproducibility of in vivo G4 peaks from three datasets, K562-rep5, K562-rep6 and HepG2-rep9, which comprise 5, 6, and 9 replicate samples, respectively (see Methods). These datasets yielded 31,130, 23,748, and 58,906 consensus regions (peaks), respectively, markedly exceeding the approximately 10,000 peaks typically reported in earlier G4 ChIP-Seq studies [7,9,15,16]. However, only a small proportion of consensus regions were supported by all replicates within each dataset, as the shared-by-all regions (i.e., peak regions consistently detected across all replicates) accounted for approximately 21%, 7.3%, and 0.5% of the total peaks in the K562-rep5, K562-rep6, and HepG2-rep9 datasets, respectively (Figure 1A). The notably low consensus in HepG2-9rep was largely attributable to one outlier replicate (GEO ID: SRR11537940), which contributed only 621 peaks (Table S1). Even after excluding this replicate and considering peaks shared by eight of the nine samples, however, the shared-by-all regions remained low at around 8%. These results indicate poor reproducibility among in vivo eG4 peaks and suggest that inconsistency across replicates has been substantially underestimated.

To assess the confidence of the called peaks, we examined their genomic distribution. The shared-by-all regions showed the strongest enrichment in promoter regions, with over 70% located in these regions across all three datasets. This finding aligns with established understanding that G4 structures are frequently associated with promoters due to their role in transcriptional regulation. We further observed a clear trend: peaks detected in fewer replicates were progressively less likely to be located in promoters (Figure 1B).

We also leveraged external G4 annotations for validation, including putative G4s (pG4s) and in vitro observed G4s (oG4s). pG4s were identified using pqsfinder [2] based on sequence potential, while oG4s were derived from G4-Seq data provided by Chambers et al. [4] Over 90% of shared-by-all regions overlapped with pG4s, and more than 70% overlapped with oG4s. Furthermore, among these peaks, a positive correlation was observed between the degree of sharing across replicates and the proportion of overlapping with pG4 or oG4 sites.

The enrichment of these peaks in promoter regions, coupled with their overlap with external G4 annotations, corroborates their biological validity. Peaks consistently detected across multiple replicates are thus more likely to represent endogenously formed G4 structures, whereas those limited to few replicates may reflect technical artifacts or stochastic noise. However, relying solely on the peak regions shared by all replicates is inadequate, as it is overly stringent and fails to retain sufficient true signals. In contrast, using the sets of occurring-at-least-once regions across replicates is overly inclusive and introduces false positives. Thus, a refined reproducibility assessment strategy for in vivo G4 data is warranted, and the distribution across different genomic regions and the support from external G4 evidence may serve as criteria for the validation of the strategy.

### 2.2. Performance of Computational Methods for Reproducibility Assessment of G4 Peaks

To evaluate the reproducibility of G4 ChIP-Seq signals across multiple replicates, we compared three computational methods based on IDR, MSPC, and ChIP-R algorithms, each employing distinct strategies for peak consistency evaluation. Given that IDR is designed for pairwise replicate comparisons, we adopted a modified approach combining results from multiple replicate pairs (see Methods). Performance was assessed using a pseudo-gold standard benchmark based on an “M of N” criterion. This framework integrated both the degree of replicate support and independent evidence from pG4/oG4 overlaps to define a high-confidence (HC) set and an artifact noise (AN) set (see Methods). Within this framework, true positives (TP) were defined as peaks overlapping the HC set, false positives (FP) as those overlapping the AN set, and false negatives (FN) as HC peaks not recovered by the method. Precision, recall, and the F1 score were subsequently calculated.

**Figure 2:**
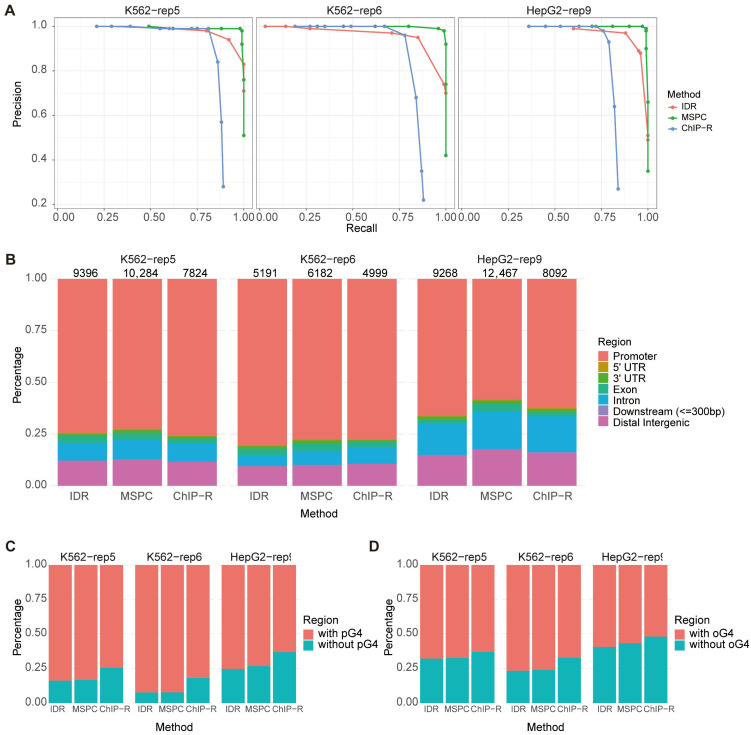
Comparison of computational methods for assessing G4 ChIP-seq reproducibility. (**A**) Precision–recall curves for IDR-, MSPC-, and ChIP-R-based methods across all replicates in the three datasets, evaluated using a pseudo-gold standard benchmark. (**B**) Genomic distribution of high-confidence peaks identified by each method at its optimal F1 score. Total numbers of these peaks are indicated above each bar. (**C**,**D**) Overlap of high-confidence peaks (called at optimal F1 score) with predicted (pG4) (**C**) and experimentally observed (oG4) G4 sites (**D**).

Precision–recall curves were generated for each method across all three datasets Figure 2A), with overall performance quantified by the area under the curve (AUC). The MSPC-based method consistently outperformed the other methods, achieving superior balance between high precision and recall. In contrast, both IDR and ChIP-R exhibited moderately lower performance, attributable in part to reduced sensitivity in identifying true positive peaks and higher false-negative rates (Table S2). The limitations of IDR likely stem from its design for dual-replicates and exclusive reliance on peak intersections, making it susceptible to inconsistencies inherent in G4 data (Figure S1). Similarly, ChIP-R assigns the lowest rank to intervals lacking peaks in a replicate prior to rank-product computation, thereby amplifying the impact of peak variability. Consequently, IDR and ChIP-R are less suited for assessing in vivo G4 data, where inter-replicate inconsistency is prominent, when compared with MSPC.

Moreover, MSPC recovered the largest number of peaks across all datasets while maintaining genomic distributions consistent with biological expectations and showing comparable levels of overlap with pG4 and oG4 sites (Figure 2B,C). These results indicate that MSPC’s ability to integrate weak but consistent signals across replicates offers a robust solution for processing noisy and heterogeneous G4 data. Based on these findings, we selected the MSPC-based approach for all subsequent analyses.

### 2.3. Optimal Number of Replicates: Three Improves Performance, Four Is Sufficient

**Figure 3:**
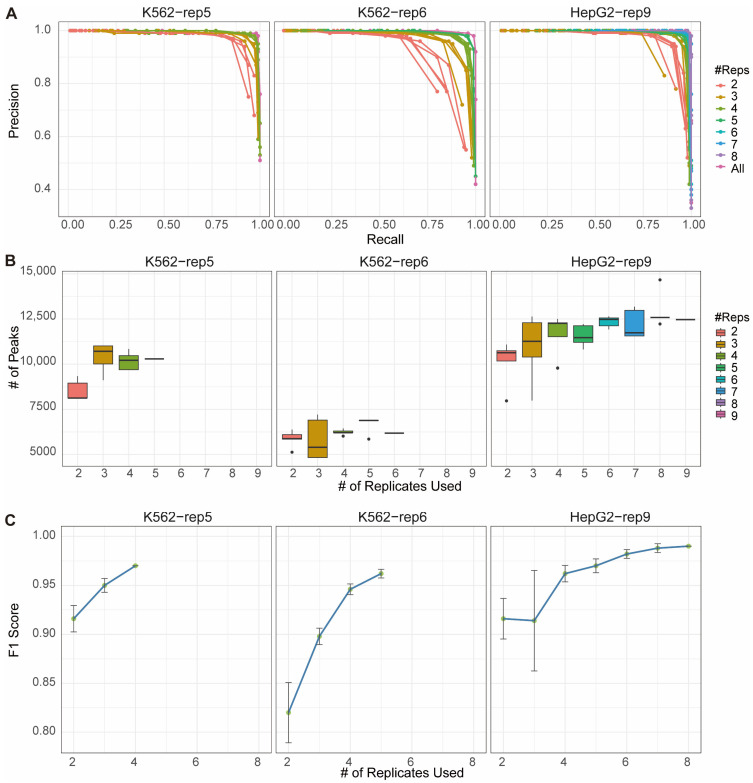
Effect of replicate number on G4 reproducibility estimated by using MSPC. (**A**) Precision–recall curves of the MSPC-based method evaluated using the pseudo-gold standard benchmark with k replicates (k=2 to N). (**B**) Number of high-confidence peaks identified at optimal F1 score across different replicate count. (**C**) Mean F1 score for each k replicate set (k=2 to N). Error bars indicate the standard deviations across trials.

A common practice in G4 antibody-based sequencing studies is the use of only two biological replicates [8,9,17], and in some cases even one, often due to experimental constraints or specific research objectives [8,9,10]. However, whether two replicates are sufficient for reliable G4 detection—particularly given the high intrinsic inconsistency of in vivo G4 data—has not been systematically evaluated. To address this, we applied the MSPC-based method to randomly selected subsets of replicates (ranging from 2 to N, where N is the total number available in each dataset) to simulate experiments with varying replicate numbers. Each subset size was tested in five independent iterations, and performance was assessed using precision–recall curves based on the same pseudo-gold standard benchmark mentioned above (Figure 3A).

A consistent trend was observed in which the reproducibility of G4 peak detection improved as the number of replicates increased, approaching the performance achieved when using all available replicates. This pattern was evident across all three datasets. Notably, the two-replicate designs not only resulted in the lowest precision and recall values but also displayed the greatest variability across repeated trials (Figure 3A). Moreover, when applying thresholds that maximized the F1 score, the two-replicate design consistently yielded the fewest high-confidence peaks (Figure 3B). These findings indicate that although two replicates may be technically feasible, they are likely insufficient for robust genome-wide profiling of in vivo G4 structures—at least within the datasets evaluated here.

In contrast, using three or more replicates led to marked improvements, yielding higher F1 scores (increasing from approximately 0.9 to 0.95, Figure 3C, Table S3) and reduced inter-trial variability (Figure 3A,C and Table S3) compared to the two-replicate design. Performance metrics from designs incorporating four or more replicates were largely comparable to those using full replicate sets, with diminishing returns beyond four replicates—each additional replicate generally increasing the mean F1 score by approximately 0.01 or less. Furthermore, the number of high-confidence peaks identified in three- and four-replicate designs was similar to that obtained with larger replicate sets (Figure 3B). In summary, utilizing at least three replicates significantly enhances the completeness and stability of in vivo G4 landscapes, while four replicates are sufficient to achieve reproducible results without substantial benefit from additional sequencing.

Notably, the aberrant F1 scores observed in the HepG2-rep9 dataset with three replicates (Figure 3C, Table S3) is attributable to sample imbalance. This imbalance stemmed specifically from the outlier replicate (GEO ID: SRR11537940) that exhibit an exceptionally low number of mapped reads (Table S1), which likely skews subsampling results. This interpretation is also supported by the correspondingly large standard deviation. Aside from this anomaly, F1 score trends remained consistent across datasets, as the replicate number increased, the effect of the outlier diminished, resulting in a corresponding increase in F1 scores and a decrease in standard deviation. This pattern again underscores the robustness of employing multiple replicates in G4 ChIP-Seq studies, even when anomalous samples are present.

### 2.4. Rescuing Experiments with Low Sequencing Depth

A further challenge in ChIP-Seq experiments is insufficient or highly variable sequencing depth across replicates [18]. Such variability often stems from both technical and biological inconsistencies throughout the experimental workflow—from immunoprecipitation to library preparation and sequencing. Although seldom highlighted in published G4 studies, empirical evidence suggests that low sequencing depth remains a common obstacle, especially for newcomers to the field. To assess whether integrating multi-replicate designs with reproducibility-aware methods could alleviate this issue, we simulated low-coverage conditions by subsampling reads from the K562-rep5 and K562-rep6 datasets to 5, 10, and 15 million mapped reads per sample, and the HepG2-rep9 dataset to 5, 10, 15, 20, and 25 million, taking into consideration their original mapping statistics (Table S1).

**Figure 4:**
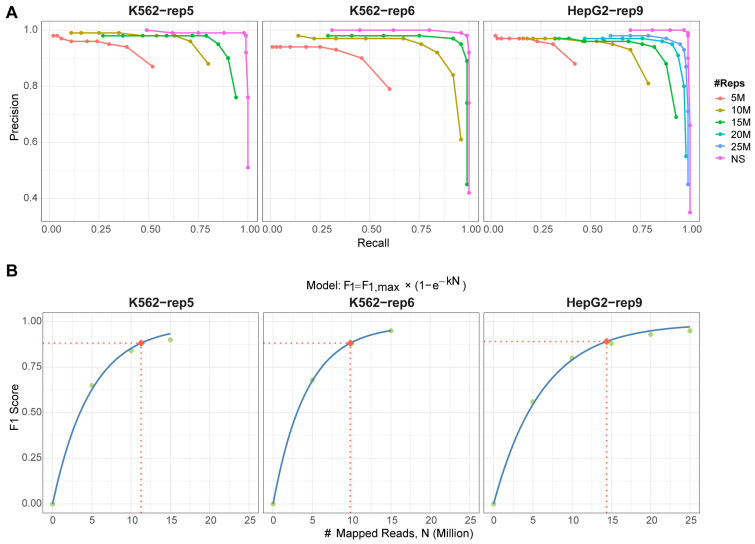
Performance of the MSPC-based method on G4 data with subsampled low sequencing depth. (**A**) Precision–recall curves of the method using all N replicates under subsampled conditions, compared to the original non-subsampled data (NS). (**B**) Non-linear fit of F1 score under different subsampled conditions, with different number of mapped reads. Green dots represent observed F1 scores, the blue curves show the fitted model $F_{1}=F_{1,max}\times (1-e^{-kN})$ where $F_{1,max}$ is the optimal F1 score generated by the original datasets, N is the number of mapped reads, and k is the parameter to be estimated. Red dots and dashed lines indicate the read amounts required to achieve 90% performance (F1 score).

We applied the MSPC-based method to each subsampled dataset using all available replicates. Increasing the sequencing depth significantly enhanced peak detection reliability (Figure 4A). Performance curves across all datasets progressively approached—yet did not fully match—those derived from the original non-subsampled (NS) data. To determine the minimal read depth required for confident peak calling, we plotted the optimal F1 score against the number of mapped reads under each subsampling scenario (Figure 4B). Using 90% of the maximum F1 score as a performance threshold, we found that approximately 10–15 million mapped reads are necessary to achieve this benchmark. This recommendation aligns with the ENCODE guidelines [19], which propose a minimum of 10 million reads per replicate for standard ChIP-seq experiments. Thus, we suggest 10 million mapped reads as an acceptable minimum for G4 ChIP-Seq studies, though depths exceeding 15 million are preferable for optimal results. Meanwhile, our findings demonstrate that incorporation of reproducibility-aware strategies with multiple replicates can compensate for the limitations imposed by low sequencing depth, at least to some extent.

## 3. Discussion

In this study, we systematically evaluated the reproducibility of G4 ChIP-Seq data by examining peak consistency across biological replicates, genomic distribution patterns, and overlap with predicted (pG4) and in vitro observed (oG4) G4 structures. Our analysis revealed substantial inconsistency in peak calls across the three representative datasets, underscoring a significant challenge for robust G4 identification and downstream functional analysis. To address this issue, we compared three computational methods—based on IDR, MSPC, and ChIP-R—using a pseudo-gold standard benchmark. MSPC demonstrated superior performance in reconciling discordant signals across replicates, while both IDR and ChIP-R were less suitable for G4 data due to their more aggressive strategies for handling low-overlap peak sets.

We further evaluated the effect of replicate number on G4 detection reliability using the MSPC framework. Although two replicates remain a common experimental design, our results indicate that three to four replicates significantly improve detection accuracy, reducing both false positives and false negatives. Beyond four replicates, however, we observed diminishing returns, suggesting that additional samples offer limited practical benefit for such studies.

Finally, we simulated low sequencing depth conditions to mimic common technical challenges in G4 ChIP-Seq workflows. We showed that leveraging multiple replicates with reproducibility-aware strategies partially offset the loss of data quality caused by low sequencing depth. Based on our findings, we recommend a minimum of 10 million mapped reads per replicate for G4 ChIP-Seq experiments, with 15 million or more reads being preferable for optimal outcomes. It remains important to note, however, the performance of such rescued data remains inferior to that of high-depth original datasets. We therefore emphasize that optimizations in wet-lab procedures should remain the primary strategy for ensuring data quality. When experimental constraints preclude such improvements, however, the use of additional replicates with reproducibility estimation offers a viable alternative for recovering meaningful biological signals.

To our knowledge, this work represents the first comprehensive effort to assess consistency across replicate samples in G4 ChIP-Seq data and to provide evidence-based recommendations for experimental design and data analysis. These findings offer practical guidance for researchers working with G4-related genomic datasets and underscore the importance of reproducibility-aware strategies in epigenomic studies.

## 4. Materials and Methods

### 4.1. Benchmark Datasets

Three G4 sequencing datasets containing more than two replicate samples were downloaded from the Gene Expression Omnibus (Table 1). K562-rep5, containing 2 biological replicates with 2 and 3 technical replicates, respectively, was from GSE107690, and K562-rep6 and HepG2-rep9, with 6 and 9 technical replicates, respectively, were from GSE145090. All the three datasets were generated by ChIP-Seq experiments with the antibody BG4. The datasets exhibit different replicate structures, from purely technical to mixed biological and technical, and the reproducibility-aware methods were tested on each dataset separately, to assess their performance in identifying robust peaks across distinct scenarios with different sources of variability.

### 4.2. Sequencing Data Processing and Peak Calling

Following the protocol suggested by Spiegel et al. [16], raw sequencing data were quality-checked and adapter-trimmed by using FastQC (v0.11.9) [20] and Cutadapt (version 2.8) [21]. Reads were aligned to the hg19 reference genome with BWA (version: 0.7.17) [22], and then filtered by using Samtools-(version: 1.17) [23]. Duplicate reads were removed with Picard (version: 2.26.4) [24]. Peaks were identified by using MACS2 (version: 2.2.7.1) [25], with $10^{-4}$ as the *p*-value threshold, which is looser than the default value and allows more candidate peaks to be identified.

### 4.3. Generation of Consensus Regions and Quantification of Replicate Support

**Figure 5:**
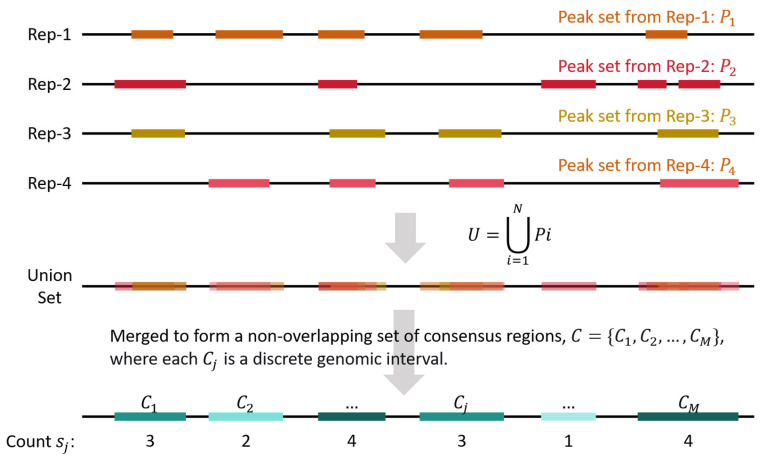
Generation of consensus regions and quantification of replicate support. The universal set of peaks was defined as the union set of peaks from all the N replicates (illustrated for N=4). This union set U was then merged to form a non-overlapping set of consensus regions. For each consensus region $C_{j}$, the number of replicates that support it (i.e., $s_{j}$) was counted.

For a specific dataset containing N biological replicates, peaks from all the replicate samples were merged and processed (Figure 5). Let $P_{i}$ be the set of genomic intervals (peaks) called from replicate Rep-i. The universal set of peak intervals is defined as the union of all peaks across replicates:

(1) $$U=\overset{N}{\underset{i=1}{⋃}}\;P_{i}$$

This union set U is then merged to form a non-overlapping set of consensus regions $C=\{C_{1},C_{2},\ldots ,C_{M}\}$, where each $C_{j}$ is a discrete genomic interval. For each consensus region $C_{j}$, its replicate support count $s_{j}$ is defined as the number of replicates in which a peak intersects $C_{j}$:

(2) $$s_{j}=\sum_{i=1}^{N}I\left(P_{i}\cap C_{j}\neq \emptyset \right)$$

where I is the indicator function that equals 1 if the condition is true and 0 otherwise.

The consensus regions were grouped by $s_{j}$, the number of consensus regions in each group was then counted, and the proportion of different groups were calculated.

The union and intersection sets were generated by using pybedtools [26].

### 4.4. Reproducibility Measurement

Five strategies were employed for measuring the reproducibility of peaks among multiple replicate samples: (1) Occurring-at-least-once; (2) Shared-by-all; (3) an IDR [12]-based method, (4) an MSPC [13]-based method and (5) a ChIP-R [14]-based method.

The first two strategies operate on consensus regions discussed above. The occurring-at-least-once strategy considers all consensus regions that appear in at least one replicate (i.e., where $s_{j}=1,\;2,\ldots ,N$), thereby maximizing sensitivity. In contrast, the shared-by-all strategy retains only those regions supported by every replicate (i.e., where $s_{j}=N$), maximizing specificity. These two methods serve as the evaluation baseline, representing the two extremes of specificity and sensitivity, respectively, and providing a reference frame for the performance of more complex methods.

IDR assesses reproducibility by comparing the consistency of peak rankings between pairs of replicates. Since IDR was designed for only dual-replicates situations, we took the union sets consisting of the reproducible peaks between any two of the replicates, where the peaks were ranked and filtered by the irreproducible discovery rate. After being filtered by a given threshold, these peaks were further merged. The rate values were used only for ranking purposes, and their implications for hypothesis testing were ignored. Different thresholds (1, 0.75, 0.5, 0.25, 0.1, 0.05, and 0.01) were tested. The version of IDR we employed was 2.0.4.2.

MSPC processes the peak lists from all replicates at once, ranking the regions containing peaks by Fisher’s combined *p*-values. Peaks shown in any of the replicates were taken into consideration (-c 1), and the algorithm was run in the “bio” form, which is a more lenient option, with the default stringency and weak thresholds. The version of MSPC we employed here was 6.0.0.

ChIP-R also processes the peaks lists from all replicates, and ranks the peaks based on a rank-product test. Peaks shown in any of the replicates were taken into consideration (-m 1), with the default cut-off value. The version of ChIP-R we used was 1.1.0.

### 4.5. Identification of pG4s and oG4s

Putative G4s (pG4s) were identified using pqsfinder (v2.0.1) [2]. In vitro observed G4s (oG4s) were derived from G4-Seq data provided by Chambers et al. [4].

The intersections of identified G4 peaks with pG4s and oG4s were accomplished by using Bedtools (v2.27.1) [27].

### 4.6. Pseudo-Gold Standard Establishment

A tiered Pseudo-gold standard was defined for each dataset based on the “M of N” principle, an empirical approach in ChIP-Seq data analysis of which effectiveness has been validated [16,18]. Additional G4 evidence, such as overlap with pG4s or oG4s, was also employed, as such information is frequently used in G4 research to support the validity of endogenous identified G4 structures [7,9,17,28]. For a N-replicate dataset, the high-confidence (HC) set consisted of peaks detected in at least 2N/3 of the N replicates ($S_{j}\geq 2N/3$) and overlapping with both pG4 and oG4 sites, which represents the most stable and robust G4 signals. Whereas the artifact noise (AN) set comprised peaks detected in only 1 replicate ($S_{j}=1$), or detected in 2 replicates ($S_{j}=2$) but missing overlapping with either pG4 or oG4 sites, which represents the most likely false positives or random noise.

### 4.7. Performance Evaluation of the Reproducibility-Estimating Strategies

The IDR-based, MSPC-based, and ChIP-R-based strategies were applied under evaluation to the three datasets, based on the pseudo-gold standard. The resulting peak sets from each method were compared against the HC and AN sets to calculate metrics such as precision, recall, and F1-score that provides a symmetric measure balancing both precision and recall. The performance of these strategies was thus quantitatively assessed. The true positives (TP) are the peaks overlapping the HC set, the false positives (FP) are those overlapping the AN set, and the false negatives (FN) are peaks within the HC set but missed by the current strategy. The metrics for performance evaluation were defined as:

(3) $$Precision=\frac{TP}{TP+FP}$$

(4) $$Recall=\frac{TP}{TP+FN}$$

(5) $$F_{1}=\frac{2}{Precision^{-1}+Recall^{-1}}$$

### 4.8. Genomic Annotation

Genomic regions were annotated by using the R package ChIPseeker (version: 1.44.0) [29]. Promoter, 5′ UTR, 3′ UTR, Exon, Intron, Downstream, and Distal Intergenic regions were annotated in the above shown priority, where Promoter regions were defined as −1 kb to +1 kb around the TSSs.

## Figures and Tables

**Table 1:** Summary of the datasets used in the study.

Dataset Name	GEO Accession Number	Cell Line	# of Replicates
K562-rep5	GSE107690	K562	5
K562-rep6	GSE145090	K562	6
HepG2-rep9	GSE145090	HepG2	9

## Data Availability

Data are contained within the article.

## Supplementary Materials

The following supporting information can be downloaded at: https://www.mdpi.com/article/10.3390/ijms26199769/s1.
