# Supplementary material for: Evaluating Reproducibility and Best Practices for Replicate Design in G-Quadruplex ChIP-Seq Studies
Source: Int J Mol Sci. 2025 Oct 7;26(19):9769. doi: 10.3390/ijms26199769 (PMC12524710; doi:10.3390/ijms26199769)
Supplement: Supplementary file 1 [file ijms-26-09769-s001.zip › SupplementaryFigures.pdf]

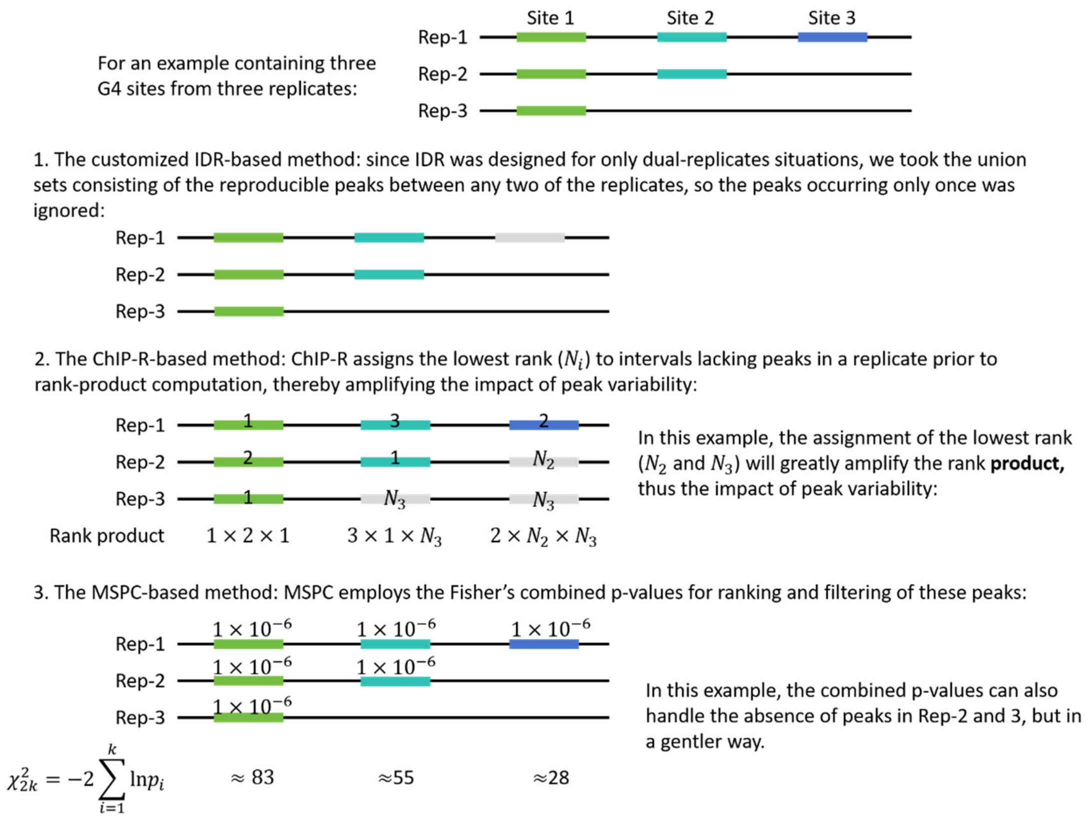

Figure S1. Comparison of the core principles and workflows of each reproducibility assessment method. Since IDR was designed for two replicates, in our customized method based on IDR, peaks occurring in only one replicate was ignored; ChIP-R assigns the lowest possible rank (equal to the total number of peaks in that replicate) to intervals where a peak is absent, thereby amplifying the impact of missing peaks during rank-product calculation; MSPC employs a combined p-value approach that also handles missing peaks, but in a more gradual and less punitive manner, which results in smaller quantitative discrepancies when peaks are absent across replicates.
